# Uncemented Cups and Impaction Bone Grafting for Acetabular Bone Loss in Revision Hip Arthroplasty: A Review of Rationale, Indications, and Outcomes

**DOI:** 10.3390/ma15103728

**Published:** 2022-05-23

**Authors:** Rocco D’Apolito, Luigi Zagra

**Affiliations:** Hip Department, IRCCS Istituto Ortopedico Galeazzi, 20161 Milan, Italy; roccodapolito@hotmail.it

**Keywords:** total hip arthroplasty, revision, acetabular bone loss, impaction bone grafting, porous cup

## Abstract

Total hip arthroplasty (THA) is increasingly performed in young patients and the number of revisions is estimated to rise over time. Acetabular osteolysis and bone loss are frequently encountered during revision and may be classified and treated in different ways. Impaction bone grafting (IBG) with morselized allograft offers a viable option. IBG was introduced over 40 years ago in combination with cemented cups, and is also used with uncemented cups. The impacted bone chips act as a void filler to restore bone stock; once incorporated they are substituted by host bone. Surgery entails assessment of the defect, acetabular preparation, preparation of the morselized graft, impaction of the graft, and cup implantation. Satisfactory medium- and long-term results have now been reported in most studies. With the advent of high-porosity cups, indications have been extended, enhancing the potential of IBG, in which primary stability of the cup to the host bone is essential for a successful procedure. Synthetic bone substitutes have also been used in combination with allogenic grafts and may extend the original technique for which long-term studies are warranted.

## 1. Introduction

Total hip arthroplasty (THA) is one of the most successful procedures in modern medicine [1]. An increase in the number of THAs performed each year and the longer life expectancy of an ageing population will result in ever more revision procedures in the future [2]. With more and more younger patients undergoing THA, it is estimated that by 2030 more than half of primary THAs will be implanted in patients under 65 years of age [3]. However, these patients are at increased risk of revision procedures over time, with a younger age at primary surgery raising the lifetime risk of revision [4]. The outcomes of revision THA in such patients are worrisome, with re-revision rates of over 25% for any reason at 10 years of follow-up [5]. The selection of biomaterials and a reduction in material wear and wear-induced debris are crucial to improve implant durability [6,7] in both primary and revision THA.

Acetabular osteolysis is one of the reasons for revision and may be accompanied by asymptomatic chronic bone loss. The degree of bone deficiency can be determined by preoperative X-ray (anteroposterior, lateral, cross-table, and Judet views) and computed tomography (CT), the latter being more accurate, whereas magnetic resonance imaging (MRI) is an adjunctive tool to assess soft tissues. Final assessment is made intraoperatively.

The most widely used classifications of acetabular bone defects are the American Academy of Orthopedic Surgeons (AAOS) Classification [8] and the Paprosky Classification [9]. The AAOS system includes the definition of cavitary (volumetric bone loss) and segmental (loss of supporting bone) defects, and grades the defects from I to V. The Paprosky system is based on an assessment of the acetabular rim as a supporting structure for the cup, together with the amount and the direction of cup migration (from grade I to IIIB and a further category of pelvic discontinuity).

Addressing a bone deficient acetabulum is often a challenge for hip surgeons. The goal is to restore hip center and joint biomechanics, while achieving stable fixation of the acetabular component. Though a minor compromise for the first two may be acceptable, firm acetabular fixation is essential for durable reconstruction and less need for revision. Strategies for restoring acetabular bone loss include the use of structural allografts, cementless hemispherical cups, oblong cups, extra-large (or jumbo) cups, modular porous augments, impaction bone grafting (IBG), reinforcement rings, and antiprotrusio cages [10].

Two types of reconstruction for major acetabular bone defects are distinguished: non-biological reconstruction in which a cup is combined with augments and/or cages (or cup-cage constructs). The metal hardware addresses the bone loss and restores the center of the hip. Custom triflanged cups and custom-made implants are reserved for extreme defects. Differently, biological reconstructions with bone grafting offer the added advantage of improving and potentially restoring bone stock for future revisions, which is particularly important in young patients [11]. Some authors do not recommend the latter procedure as the first choice because it may be technically demanding and time-consuming as compared with non-biological techniques, with the risk of graft resorption and infection. Other reasons are the lack of suitable graft material and biologic fixation in cemented IBG [12]. While cementless IBG provides potential biological fixation when combined with a technique that most surgeons may be more familiar with (e.g., hemispherical cups), technical skills remain essential for a successful procedure. Indications for cementless IBG can be extended to major bone defects, in which last generation acetabular implants have redefined the need for pre-existing bone stock and minimum contact between the implant and the host bone to obtain primary fixation.

The most important factor in long-term fixation is bone stock [13]. The use of uncemented cups with adjunct screw fixation is a well-established method for acetabular revision when bone stock is deficient [14]. However, it might not suffice in intermediate and major bone loss, in which the use of uncemented cups can be combined with IBG to fill the defects and achieve better and more durable reconstruction and bone restoration.

The IBG technique was first described more than 40 years ago. Most studies are focused on cemented IBG, with or without mesh. Though not as well established as the cemented technique [15], IBG has been used in cementless revisions, with promising results in combination with new materials. This review summarizes the rationale and technique of IBG with cementless cups in revision THA, provides technical notes from our practice, and describes the results obtained with the technique.

## 2. History of IBG

The conception and the development of IBG are closely related to the use of cemented cups. In 1975, Hastings and Parker first described IBG using cemented cups for protrusio acetabuli in rheumatoid patients [16]. In 1984, almost 10 years later, the technique was popularized for primary and revision THA by Slooff et al. from the Netherlands, who reported the results of 43 hips in which graft union was radiographically confirmed between 2 and 4 months after surgery [17].

In the original procedure, a metal mesh was used to cover the graft and limit cement penetration. This was soon abandoned because it hindered graft incorporation and did not add any relevant mechanical stability, whereas metal meshes secured with screws remained part of the technique to close the segmental defects of the acetabular wall or on the peripheral rim. Later, the same group reported the outcomes of 88 revisions using aseptic loosening as endpoint, with a 94% survivorship at a mean follow-up of 11.4 years (minimum 10 years) [18]. These results were confirmed also in the long term (20 to 25 years), with an 87% survivorship [19].

Similar results have been reported by other groups [20,21,22], with favorable outcomes in patients under 55 years [23,24,25]. Higher failure rates were associated with larger defects (Paprosky type III) [26,27], however, and less experienced surgeons found the technique difficult to perform, even when assisted by a more experienced colleague [26]. Moderate bone defects (20 mm in depth and without multiple segmental defects) were considered to be predictors for better results [28]. Over time, the original technique has been refined and metal augments and structural allografts are now used in modern reconstructions with cemented components.

## 3. Rationale of IBG and Cementless Cup

Different from its historical development in combination with cemented cups, IBG and cementless cups offer several advantages. The rationale lies in the biological changes taking place in the graft during the postoperative period. In the treatment of acetabular defects, IBG is a biological void filler and restores bone stock once it is incorporated and substituted by host bone. Theoretically, an autograft, being osteoinductive and nonimmunogenic, ought to be the best biological option for bone restoration [29], but its major limitations in acetabular reconstruction are scarce availability, donor-site morbidity, and time-consuming collection. In contrast, morselized grafts from frozen heads maintain an adequate surface/volume ratio as compared with strut grafts, which means higher remodeling properties with less necrotic residual tissue; they can be used to fill defects in a “personalized” procedure and reach good mechanical properties when properly impacted, as noted for cemented IBG [17].

Histological data show that a creeping substitution takes place, with vascularization and incorporation of the impacted bone graft [30]. A healing scenario partly mimicking bone fracture healing starts from the periphery with partial osteoclastic resorption of graft trabeculae and application of living bone to allograft fragments. The graft fragments are gradually remodeled with progressive vascular ingrowth, until the graft is almost completely incorporated [30,31,32]. This process begins in the first weeks after surgery and continues for years after the operation. The extent of graft incorporation cannot be predicted accurately by postoperative radiographs [31]. Though the fibrous tissue may not be completely replaced by new bone tissue in some cases, the clinical result does not appear to be affected, at least in femoral cemented IBG [33].

An essential point to bear in mind during surgery is that primary stability of the cup is the cornerstone for a successful procedure. Unlike IBG with cemented cups, where cup stability depends on a firm bed of impacted bone, in cementless implants, the cup can migrate and eventually fail if its stability relies only on a graft that is being resorbed [34]. While bone ingrowth is promoted by the bleeding host bone, ingrowth through a morselized graft probably takes years, if ever, to occur [13]. This is why sufficient cup-host bone contact is essential. Though this mean a limitation of the technique, the cup features make the difference; high failure rates in the past were due to inadequate cup design and surface finishing [35]. In the last two decades, tantalum and titanium high-porosity cup surface finishing by different additive technologies have changed the history of acetabular revision. Primary stability of the cup to the host bone of 50% was initially the cutoff for better results [36], whereas 25–30% cup-host bone contact is now considered to be acceptable for long-term fixation, if primary stability is achieved with a good rim fit and a contact area on the dome and the posterior column with the addition of multiple screws [37,38].

## 4. Surgical Technique

**Figure 1:**
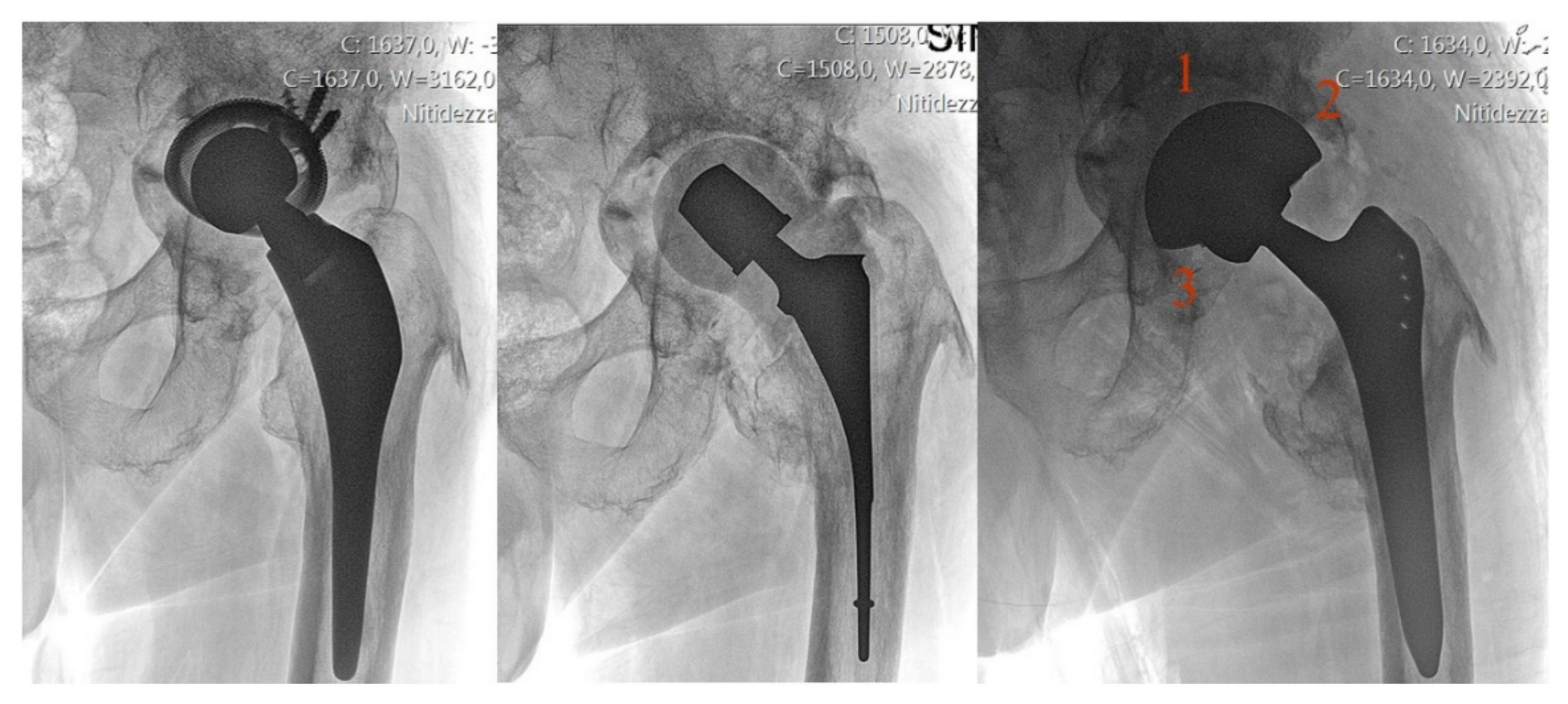
Three-point contact support for the cup: Point 1 antero-superior; point 2 postero-superior; point 3 postero-inferior. Points 1 and 2 are provided by the ilium and point 3 by the ischium.

The first part of the operation includes exposure of the acetabulum, as done in any revision. The cup is removed while sparing as much viable bone as possible, and the same is done for the cement, if present. Any acetabular pseudomembrane is debrided and the acetabulum is curetted untill bleeding or to the living bone before reaming. The first goal is to obtain press-fit contact of the cup by maximizing host bone-shell contact without causing further bone loss during reaming. The second is to achieve three-point contact support: superiorly, on the antero-superior and the postero-superior ilium, and postero-inferiorly on the ischium, with at least one, better if two, opposing portions of the acetabular wall (Figure 1) [15].

**Figure 2:**
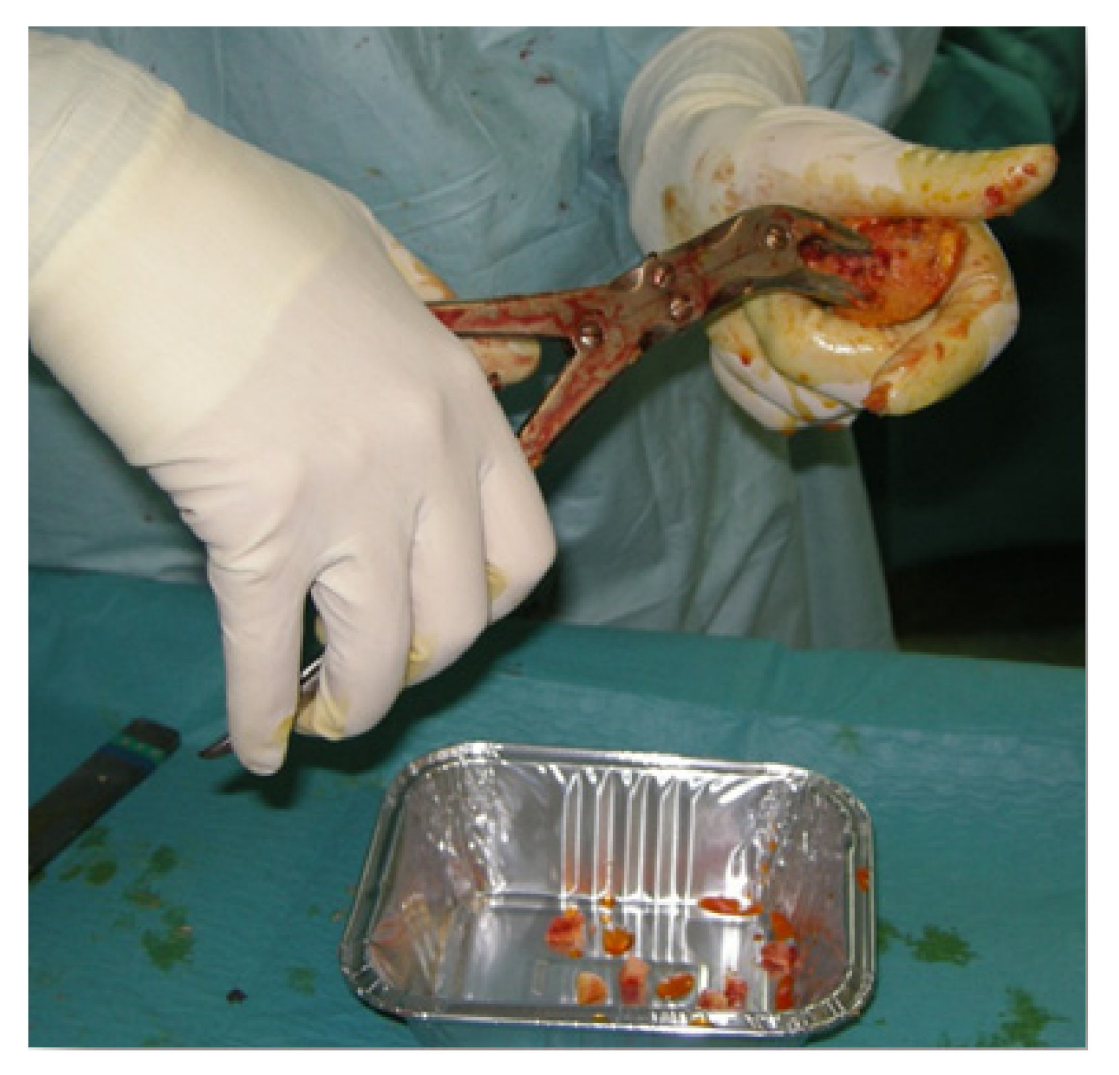
Preparation of morselized bone from an allogenic femoral head.

A trial component is put in place to check the seating and to determine the amount of morselized bone needed to fill the defects and the sections to be filled. One or more fresh-frozen (within 24 h of harvesting), non-irradiated, allogenous femoral heads are used as graft. The bone is morselized manually with a bone nibbler (Figure 2).

**Figure 3:**
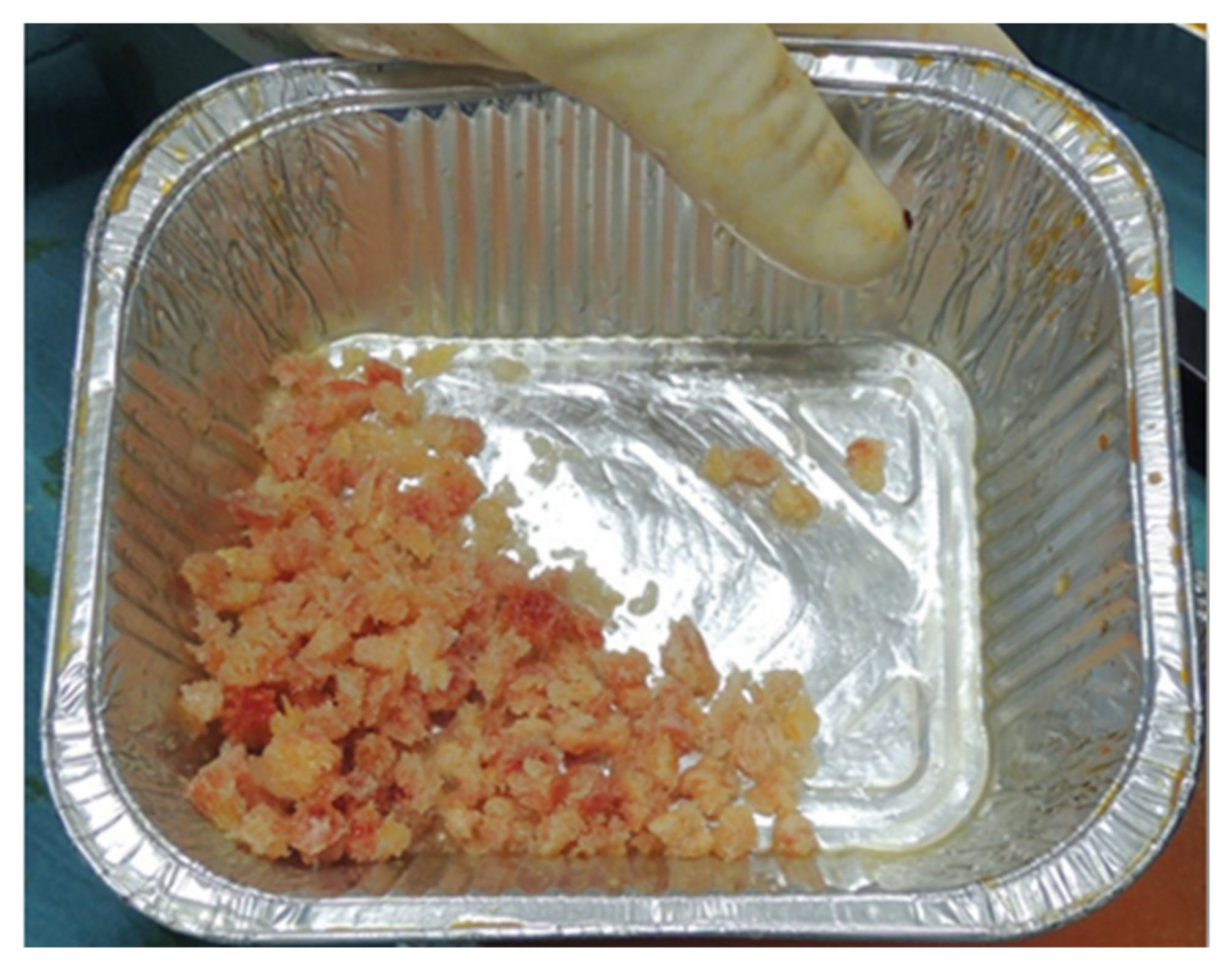
Bone chips after repeated washing to eliminate blood and bone marrow fat.

The cancellous bone chips range in size from 3 to 5–10 mm^3^. Based on synthetic models, large bone chips washed prior to impaction are mechanically superior to small chips [39], and larger chips are recommended by experienced surgeons [27]. Moreover, careful washing reduces the risk of bacterial contamination [40] and host immunologic response (due to removal of bone marrow fat) and promotes bone graft incorporation [41]. The bone chips are repeatedly washed with saline until bone marrow fat and residual blood traces are removed (Figure 3).

**Scheme 1:**
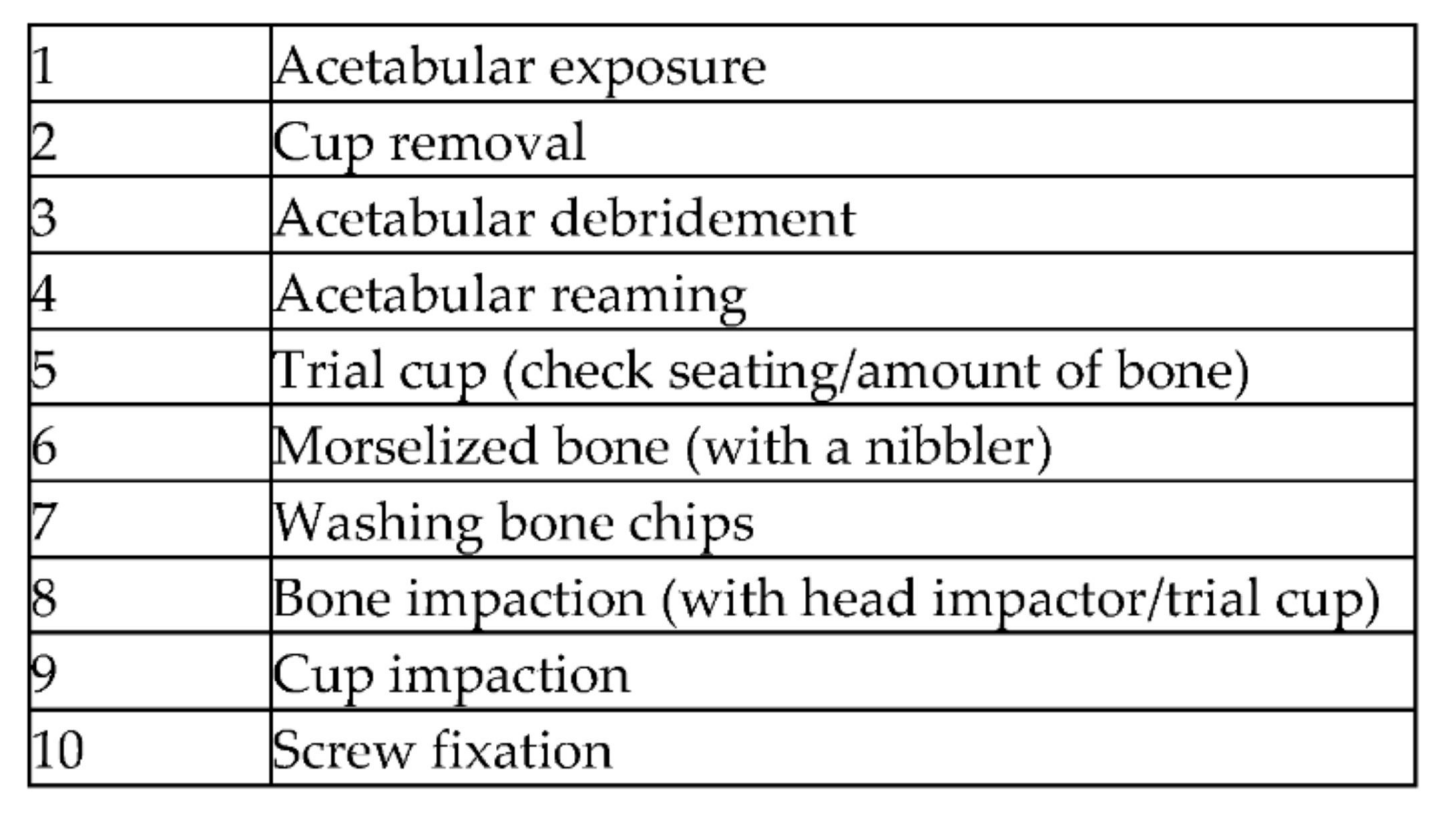
Outcomes of IBG and cementless cup.

The morselized graft is then impacted into the bone-deficient acetabulum. This may be done using the original technique, which involves increasing the diameter head impactor to firmly impact the graft or using the trial component (when appropriate) in the last step or reverse reaming to create a hemispherical cavity [13,15,42,43,44]. Experienced surgeons advise against reverse reaming [27,45], which can reduce graft stability. Stability is achieved with a solid wall of strongly impacted bone grafts and without the graft moving under manual pressure. Firm impaction promotes bone formation and enhances implant stability, acting similar to “customized” bone augmentation to fill the defects between the part of the host bone not in direct contact with the shell and the cup surface. The revision shell (1–2 mm larger than the last reamer diameter) is then impacted. It is fixed on the host bone and the graft is put under pressure. Most surgeons secure the cup with screws, irrespective of perceived stability. Since primary stability of the cup is crucial for medium- and long-term success, screws should be used only as adjuvant fixation, whereas primary cup support should rely on native acetabular bone, with effective primary stability achieved even without screws (Scheme 1). In second-stage revision for infected THA, the morselized graft can be added with antibiotics for local antibacterial delivery [46].

The results of IBG with cementless cups were initially associated with consistent failure [47,48,49] due to inadequately designed acetabular sockets. The technique was not recommended for treating larger defects because of the persistent belief that a 50% host bone contact was necessary for ensuring initial stability and bone ingrowth [50]. For the same reason, IBG was usually associated with cemented cups until the advent of technical improvements and highly porous cups. Moreover, the technique benefitted from the lesson learned from the cemented IBG technique, i.e., that firm impaction of the morselized graft is essential to fill the defects.

Case series have reported outcomes with the technique. Jasty reviewed 19 jumbo acetabular reconstructions for major acetabular bone loss (a 4 cm or larger defect in the medial wall and loss of the anterior column) using IBG at 10 years of follow-up, and reported that all the grafts had united and that the medial wall was reconstituted in all but one patient with pelvic discontinuity, who developed aseptic loosening [51]. Garcia-Cimbrelo reported four cases of graft resorption and seven cases of definite cup loosening in 42 patients with Paprosky type II acetabular defect at a mean follow-up of 8 years [50]; the study underlined the concept of 50% contact with host bone. Later, Palm et al. described promising results of extensive allograft impaction and hydroxyapatite-coated cup with supplementary screw fixation in 87 hips at 9 years (7–11) of follow-up, with a 90.5% survival rate and revision for any reason as endpoint, and a 94% survival rate, with revision for aseptic loosening as endpoint [14]. In contrast, Lazarinis et al. found no evidence based on Swedish Hip Arthroplasty Register data that hydroxyapatite coating improved performance in acetabular revision [52]. Lee and Nam reported the results of 71 hips at a mean 12-year follow-up with a 95.8% survival (Paprosky grade I to IIIB defects) [13]. Bilgen et al. reported on two re-revisions at a mean follow-up of 8 years in 15 patients undergoing IBG with cementless cups in which 100% or virtually 100% of the component contacted only with the allograft [43]. A recent study by Perlbach et al. reported an implant survival rate of 96.3% for aseptic loosening in 370 patients after 10 years and a 92.8% rate after 15 years [15] (Table 1).

Ultraporous cups have dramatically changed the perception of the amount of host bone needed to obtain stability. Good results have been reported in contained defects treated with trabecular cups having 50% or less (19% on average) contact with host bone [36,37,44]. In addition, while the percentage of host bone contact is not an independent risk factor for cup migration and loosening, primary stability is a predictor for migration (but not for failure) in the midterm [38]. These more flexible cups allow for a more “iso-elastic” loading of bone and reduce stress shielding, therefore, explaining the increased periacetabular bone density as compared with stiffer cup designs on quantitative CT scans [53]. This might be useful to prevent bone graft resorption, especially when larger volumes of IBG are used [34].

The availability of ultraporous augments further enhanced the potential of IBG and redefined the acetabular rim for containing the graft. Augments can offer more predictable mechanical performance than mesh, with greater stability and ease of use. Borland et al. reported satisfactory clinical and radiographic results using tantalum augments in 24 patients (15 Paprosky grade IIIA defects and 9 grade IIIB defects), with a low failure rate at a median follow-up of 5 years (one patient with fractured augment) [54]. No revisions at a mean follow-up of 3 years were reported by Gill et al. in 15 hips treated with augments for from grade I to IIIB defects [55]. Gehrke et al. reported promising results in patients with 28 type IIB and 18 type IIIA Paprosky defects [56].

Synthetic bone substitutes may avert the risk of disease transmission and immune response associated with allogenic grafts. Aulakh et al. compared the results of patients receiving an allograft alone (n = 42) and others receiving a 50/50 mix of hydroxyapatite and allograft (n = 23) at 13 years and reported an 84% survival rate for the allograft group and an 82% rate for the mixture group [57]. McNamara et al. reported the results of THA using a 1:1 mixture of frozen, ground and irradiated bone graft and Apapore 60, a synthetic bone graft substitute, at a mean follow-up of 5 years in 50 hips with a 100% survivorship rate (30 acetabular grafts showed evidence of incorporation, 10 acetabular grafts showed radiolucent lines, and 2 acetabular components migrated initially before stabilizing) [58]. In the longest survivorship report of IBG with morselized bone and hydroxyapatite substitutes in 47 THAs [59], Abdullah et al. stated that 11-year survivorship, function, and pain were excellent, though radiological findings of lysis in eight cases and migration in four cases might be of concern for the immediate future and need close monitoring. Based on their experience with morselized bone graft material from fresh frozen heads manually prepared during surgery, the authors considered it to be the technique of choice when bone from the local bone bank was available.

The present narrative review has several limitations. Selection bias might have influenced our findings, though we did not exclude studies based on their quality or conclusions. Our aim was to provide surgeons who are interested in the technique with a concise overview of uncemented IBG. The bulk of the literature on impaction grafting was not included because it focused mostly on IBG with cemented cups. We also did not perform a quantitative analysis of the data due to the design and the heterogeneity of the studies. Finally, since the topic was uncemented IBG, other techniques to address acetabular bone loss were beyond the scope of this review, which was not intended to demonstrate the superiority of this technique over others.

## 5. Conclusions

Among currently available techniques for repairing bone defects during revision THA, IBG offers an effective option for managing bone loss and biological reconstruction, while restoring hip biomechanics and bone stock, the latter being of importance for future revisions. Older studies have reported discouraging findings [47,48,49] as compared with the original (cemented) IBG technique [19,20,21,22]. More recent data has supported the use of cementless IBG for bone defects and reported good implant survival in the medium term, with encouraging data for the long term [13,14,43,50,51], comparable or superior to the original technique in some cases. High-porosity cups are probably the game changer in this scenario [44], whereas porous metal augments can help to address complex defects [54,55,56] and further extend indications for the type of bone defects to be treated. When properly and firmly impacted, morselized grafts improve the primary stability of the implant and can be added with antibiotics for local delivery.

This review reports the rationale and the technical key points to obtain good long-term clinical outcomes, as reported in the literature for this technique. A surgeon’s experience and ability to identify the types of patients and defects that can benefit from IBG also play major roles in obtaining reproducible results. Further research in multicenter comparative studies is desirable.

## Figures and Tables

**Table 1:** Summary of isolated IBG outcomes.

Author	Defect	Hips (n)	Follow-Up (years)	Survivorship
**Jasty et al.**	>4 cm Medial wall defect and loss of anterior column	19	10	1 Aseptic loosening
**Garcia-Cimbrelo**	Paprosky II	42	8	7 Loosening, 4 graft resorption
**Palm et al.**	Gustilo & Pasternak I–IV	87	9	91% For any reason, 94% for aseptic loosening
**Lee and Nam**	Paprosky I–IIIB	71	12	96%
**Bilgen et al.**	Paprosky II–IIIB	15	8	87%
**Perlbach et al.**	AAOS I–IV	370	15	93%

## Data Availability

Not applicable.
