# Correction: D’Apolito, R.; Zagra, L. Uncemented Cups and Impaction Bone Grafting for Acetabular Bone Loss in Revision Hip Arthroplasty: A Review of Rationale, Indications, and Outcomes. *Materials* 2022, *15*, 3728

**DOI:** 10.3390/ma15165683

**Published:** 2022-08-18

**Authors:** Rocco D’Apolito, Luigi Zagra

**Affiliations:** Hip Department, IRCCS Istituto Ortopedico Galeazzi, 20161 Milan, Italy

The authors wish to make the following correction to this paper [1].

**Missing Funding**

In the original publication, **the supporter and funder, Italian Ministry of Health—Ricerca Corrente 2022**, was not included. The authors apologize for any inconvenience caused and state that the scientific conclusions are unaffected. This correction was approved by the Academic Editor. The original publication has also been updated.
